# Feasibility and correlations of smartphone meta‐data toward dynamic understanding of depression and suicide risk in schizophrenia

**DOI:** 10.1002/mpr.1825

**Published:** 2020-04-25

**Authors:** Philip Henson, John Torous

**Affiliations:** ^1^ Department of Psychiatry, Beth Israel Deaconess Medical Center Harvard Medical School Boston Massachusetts USA

**Keywords:** duration, meta‐data, smartphone, suicide, surveys

## Abstract

**Objectives:**

We investigate whether meta‐data, specifically duration of responses to smartphone‐delivered surveys, is correlated to elevated scores on the depression assessment PHQ‐9 as well as the specific item around self‐harm (item 9).

**Methods:**

In this observational study, we recruited 92 smartphone‐owning adults (≥ 18) with schizophrenia (45) and healthy controls (43). We installed an open‐source smartphone app called mindLAMP to collect survey results and latencies (response times) over a period of 3 months. Surveys were scheduled for twice a week, but participants were instructed to take the surveys naturally as much or as little as they wanted. A total of 1,218 PHQ‐9 surveys were completed across all participants over 3 months.

**Results:**

A total of 75 participants (39 with schizophrenia and 36 healthy controls) completed both the initial visit and follow‐up, as well as provided at least one self‐reported PHQ‐9 survey through the app. We found that depression symptom severity and response latencies were correlated for both individuals with schizophrenia (Spearman's *ρ* = .22, *p* = .037) and healthy controls (Spearman's *ρ* = .58, *p* < .001). Participants with schizophrenia scored higher (more severe) and took longer for every item of the PHQ‐9 when compared to controls (*p* < .05 for each item). Item 9 response value and latency was slightly correlated for participants with schizophrenia (Spearman's *ρ* = .086, *p* = .035) but was not significant for controls (Spearman's *ρ* = .036, *p* = .37).

**Conclusions:**

Meta‐data revealed group differences between individuals with schizophrenia and healthy controls based on individual depression symptoms completed on a smartphone. Correlation between suicide specific question latency and severity for participants with schizophrenia but not for controls indicates the clinical potential and need for further research.

## 1 INTRODUCTION

As suicide rates in the United States continue to rise, there is an urgent need to better identify those at risk and offer preventive services. Realizing that dynamic risk factors for suicide cannot easily be quantified in a routine clinical assessment, there has been increased interest in digital tools like smartphones to better capture the real‐time and movement‐to‐moment changes that may better define risk. Already impressive research is using smartphone‐based surveys to assess risk factors numerous times per day and understand how symptom variability impacts outcomes (Kleiman et al., 2018). In addition to this active data collection by patients, smartphones can also capture passive data with signals like geolocation, sleep, and steps offering functional data to inform how environment and activity interplay with risk (Torous et al., 2018). Clinically, the value of active and passive data to provide new subjective and objective data, respectably, on suicide risk is clear. However, there is a third source of data that may offer further value toward risk assessment called meta‐data.

Meta‐data related to how people interact with their screens may offer an actionable data source to inform suicide risk. As an example, consider a person answering “zero” on item 9 of the PHQ‐9, an evidence‐based measure of depression severity (Kroenke, Spitzer, & Williams, 2001), indicating they have had no thoughts of self‐harm or suicide in the last 2 weeks (Jo, Na, & Kim, 2018). How would clinical assessment of risk change if it is now possible to know the person answered the prior eight items in a total of 20 s but spend 2 min before picking an answer to the question about suicide? While spending more time on that particular question does not inherently mean a person is at greater risk, it does offer a potentially useful opportunity for further discussion. Already, this method of examining response times (latencies) has been utilized in computer‐administered subjective well‐being (SWB) surveys as a means to identify poor quality data (Yan, Ryan, Becker, & Smith, 2015) and touchscreen PHQ‐9 to separate respondents by age and score (Fann et al., 2009). But, to date, few studies have examined how meta‐data composed of these latencies when completing suicide risk assessment on a smartphone may be able to augment risk prediction.

While the process of suicide is complex and still poorly understood, there is already evidence that those taking a longer time to respond to questions about suicide may be at elevated risk (Deisenhammer et al., 2009). Changes in survey latencies could also reflect alterations in neurocognition related to common pathways for suicide risk, especially related to decision‐making (Westheide et al., 2008). While most research on neurocognition and suicide is based on lengthy but validated in‐person assessments (Pu, Setoyama, & Noda, 2017), smartphone‐derived meta‐data as a proxy for executive functioning requires no additional time, although it lacks a research foundation.

Meta‐data related to survey latencies are generated automatically when a user completes a smartphone assessment and both response value and response time are stored. Unlike with passive data where geolocation or call/text logs may be captured, there are fewer ethical implications with meta‐data as it is not identifiable or easily subject to misuse. There are already smartphone apps that install external keyboards and thus capture every keystroke whether the person is using the app or not, but the public has raised privacy and ethical concerns around this type of meta‐data capture (Rooksby, Morrison, & Murray‐Rust, 2019). The meta‐data we explore in this paper is different as it is related only to the timing of survey and cognitive test responses as captured when a survey or cognitive test is being actively taken by the user.

To explore the potential clinical utility of this meta‐data, we compared how app‐based latencies on item 9 of the PHQ‐9 compared to latencies for items related to depression and how these were related to in‐person assessments. Given that suicide and suicidal ideation is a relatively rare event, we explored data from a cohort of patients with schizophrenia where lifetime the rate of suicide is approximately 5% (Hor & Taylor, 2010; Palmer, Pankratz, & Bostwick, 2005) and prevalence of suicidal ideation is about five times that of the general population (Breier, Schreiber, Dyer, & Pickar, 1991; Nock et al., 2008). We hypothesize that latencies across survey types are associated with each other, that latencies on PHQ‐9 will be greater for individuals with schizophrenia than for healthy controls, and that longer latencies on item 9 will correlate to higher symptom severity for that item for both individuals with schizophrenia and healthy controls.

## 2 METHODS

### 2.1 Participants

A total of 92 smartphone‐owning adults from the Boston area were enrolled in the LAMP smartphone study (IRB 2017P‐000359, September 27, 2019) from August 22, 2018, to February 13, 2019. A recruitment goal of 50 patients was set after a successful pilot study with 17 participants with schizophrenia (Barnett et al., 2018). Among the 92 participants (Table 1), 75 (39 with schizophrenia, SZ and 36 health controls, HC) completed both in‐person visits and provided at least one self‐reported PHQ‐9 on the mindLAMP app (Torous et al., 2019).

**TABLE 1:** Participant demographics. Seventy‐five smartphone‐owning study participants from the greater Boston area participated in this 3‐month smartphone study

	HC (*n* = 36)	SZ (*n* = 39)	*p*
Age	31.97 (16.65)	37.00 (14.86)	.193
Gender			1
Male	17 (47.2%)	20 (55.6%)	
Female	19 (52.8%)	19 (44.4%)	
Race			<.001
American Indian or Alaskan native	0 (0.0%)	4 (10.5%)	
Asian	25 (69.4%)	1 (2.6%)	
Black or African‐American	3 (8.3%)	11 (28.9%)	
Multiracial or other	2 (5.6%)	1 (2.6%)	
White Caucasian	6 (16.7%)	21 (55.3%)	
Education			<.001
Four‐year college graduate or higher	30 (83.3%)	14 (35.9%)	
High school graduate/GED	3 (8.3%)	11 (28.2%)	
Some college	3 (8.3%)	11 (28.2%)	
Some high school	0 (0.0%)	3 (7.7%)	

Abbreviations: HC, health controls; SZ, schizophrenia.

### 2.2 Data collection

**FIGURE 1:**
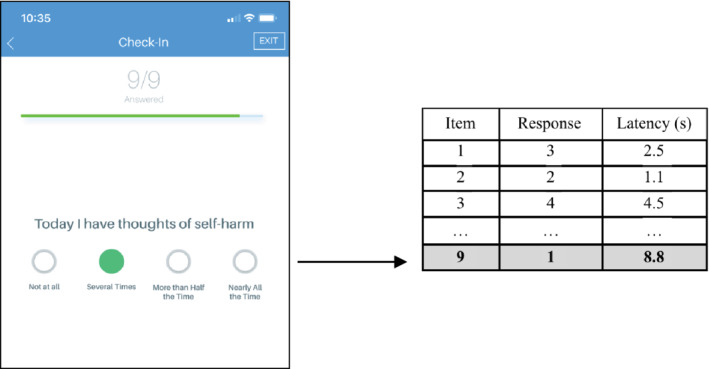
Screenshot of the PHQ‐9 within the LAMP app and example of recorded data. The app auto‐scrolls to facilitate survey completion and automatically records response values (from 0 to 4) and time taken for each response (latency)

Mood and cognition were collected as part of the LAMP smartphone study, which has been previously reported on Liu, Henson, Keshavan, Pekka‐Onnela, and Torous (2019), Torous et al. (2019). In‐person assessments at the Massachusetts Mental Health Center (Boston, MA) were collected at the initial visit and final visit after 90 days. Participants completed PHQ‐9 with a clinician and a cognitive functioning assessment with a validated iPad version of the Brief Assessment of Cognition in Schizophrenia (BACS, SZ only) (Atkins et al., 2017). Throughout the study, mood was collected on all participants' smartphones twice a week for 3 months via PHQ‐9 taken in the LAMP app (Figure 1). PHQ‐9 questions were identical between the paper and pencil survey and smartphone survey with the exception that in person, participants are asked to reflect over the past 2 weeks, whereas in the app, participants are asked to reflect just on that day. Participants were notified on their smartphones whenever a new survey was available which remained on the app for participants to complete at their own discretion. The phone began the timer for latency once the survey was opened via the notification, and ended the timer once submit was pressed at the end of the survey. Each item response value is associated with a single latency value. Participants were not compensated for responding to any smartphone surveys, although were paid for in‐person clinical assessments. Participants were notified that if they indicated an elevated risk of suicide on the app, the app would direct them to resources, but their responses would not be monitored or responded to in real time. All participants were also offered other surveys related to anxiety (GAD‐7) as well as questions related to social functioning, sleep, and psychosis‐related symptoms.

### 2.3 Analysis

All analyses were performed using the R programming language (version 3.5, https://www.r-project.org/). Several relationships among the mood and cognition results were investigated: (a) comparison of smartphone‐collected survey scores and latencies within SZ and HC, (b) comparison of paper and pencil PHQ‐9 score to smartphone PHQ‐9 scores using linear regression, (c) specific investigation of item 9, and (d) effect of cognition on survey latencies. Correlations were conducted using Spearman's rank correlation coefficient and *p*‐values were adjusted using the false discovery rate method.

### 2.4 Results

**FIGURE 2:**
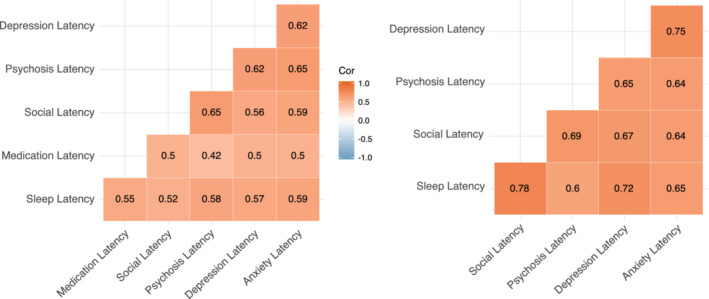
Correlation heatmap of self‐reported survey latencies with schizophrenia on the left and health controls (HC) on the right. Note all correlations are significant apart form the two marked with “X” for HC related to psychosis symptoms

We assessed correlations between latencies of different smartphone‐administered surveys (PHQ‐9 (depression), GAD‐7 (anxiety), sleep, psychosis, and social) to ensure that latency magnitude was comparable across survey domains. All survey latency correlations were significant (*p* < .05) for both SZ and HC except for two associations with psychosis latency in HC, who were screened for not having psychosis prior to enrollment (Figure 2).

When comparing the correlation between total score and latency of responses, depression showed the highest significant correlation compared to other surveys in both SZ and HC at *ρ* = .22 (*p* = .037) and *ρ* = .58 (*p* < .001), respectively (Table 2).

**TABLE 2:** Survey score versus survey latency. Correlation (Spearman's rho) between average survey score and latency was calculated for schizophrenia (SZ) and health controls (HC)

	Patients		Controls	
Survey type	Rho	*p*‐Value	Rho	*p*‐Value
Sleep	−0.21	.44	−0.36	<.001
Social	−0.15	<.001	0.34	<.001
Psychosis	0.11	.66	0.29	.0033
**Depression**	**0.22**	**.037**	**0.58**	**<.001**
Anxiety	0.027	.070	0.47	.070

*p*‐values are given direct in table.

**FIGURE 3:**
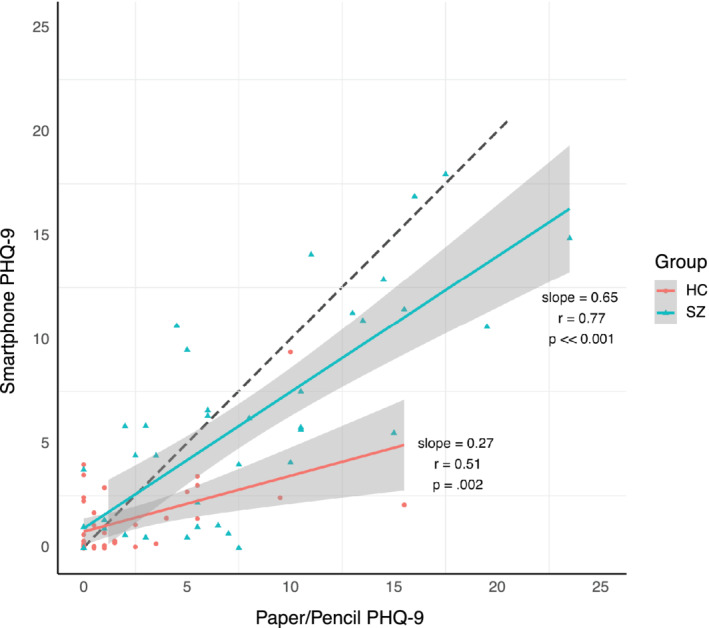
In‐clinic depression assessment with the PHQ‐9 versus smartphone assessments with the PHQ‐9 offered outside of the clinic. The same score on both is reflected by a hypothetical unity line (dashed line) and scores deviating from the line indicated differential reporting of symptoms

Figure 3 demonstrates a visual comparison between paper and pencil PHQ‐9, administered with a clinician, and smartphone‐delivered PHQ‐9, completed outside of study visits, revealed that both the SZ and HC populations underreport symptoms in the app compared to in the clinic (slope = 0.65 and 0.27, respectively).

**FIGURE 4:**
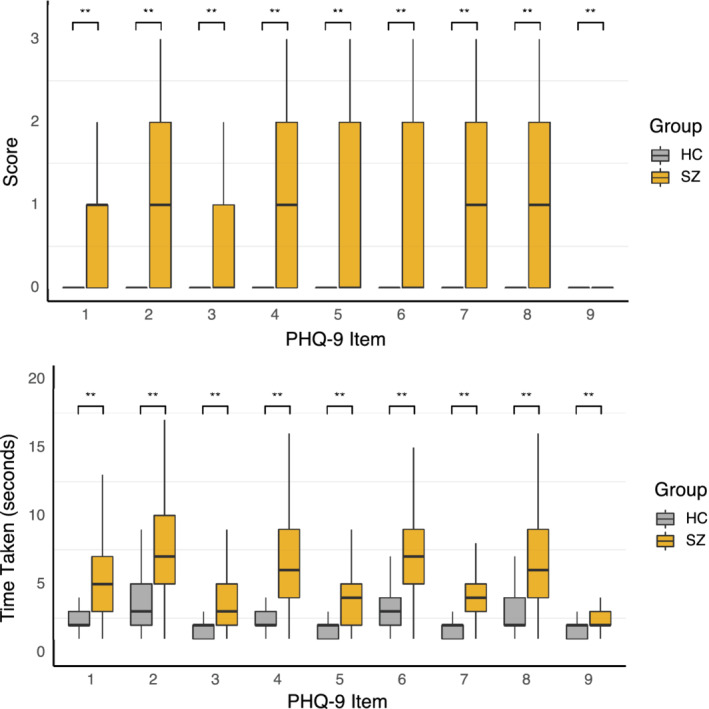
PHQ‐9 score (top) and latency (bottom) group differences. Note the health controls scores are ~0 for scores in the top figure as this sample was selected for lack of mental illness. Score latencies were lower for controls across all items as compared to patients. ** indicated *p* < .05 for each item whether examined by score or latency

The nine individual items of the PHQ‐9 were separated and analyzed for score and latency in both SZ and HC. Controls completed each of the nine items more quickly and thus had lower latencies and also reported lower scores compared to patients in response to all nine items (*p* < .001 for each item) (Figure 4). For both SZ and HC, paired *t*‐tests found that item 9 latency was significantly lower than the combined average of items 1–8 by 3.3 s (*p* < .001) and 1.3 s (*p* < .001), respectively.

We examined the correlation between the score on each item of the PHQ‐9 and latency for that item, with results shown below in Table 3. Correlations were generally low for all items, although of note not significant for item two (feeling down, depressed, or hopeless) in SZ and item 9 in HC.

**TABLE 3:** **Correlations between score and latency for each item of the PHQ‐9**. Correlation value represented is Spearman's rho

Item	Rho (SZ)	*p* (SZ)	Rho (HC)	*p* (HC)
1	0.0834	.04	0.432	<.001
2	−0.0066	.87	0.191	<.001
3	0.2447	<.001	0.317	<.001
4	0.1165	.004	0.365	<.001
5	0.1366	<.001	0.267	<.001
6	0.1431	<.001	0.197	<.001
7	0.1515	<.001	0.311	<.001
8	0.1531	<.001	0.239	<.001
9	0.0864	.035	0.036	.372

Abbreviations: HC, health controls; SZ, schizophrenia.

We also used results from the BACS during the first and second study visits to control SZ for cognition, with results displayed in Appendix A.

## 3 DISCUSSION

Meta‐data related to survey latencies of smartphone‐based surveys of depression and suicide offer novel information that may be of utility in assessing risk. Compared to other surveys (sleep, social, psychosis, anxiety), only the PHQ‐9 assessing depression showed a significant positive correlation between total score and latency of response (see Table 2). Focusing on item 9 related specifically to suicide, there was a small but significant correlation between score and latency in SZ but not HC (see Table 3). The overall lower severity of symptoms reported in both cohorts via the smartphone app as compared to in‐clinic assessments suggests clinical nuance in using the data.

Our results of lower mean symptom reporting of depression via the smartphone app as compared to in‐clinic assessments (Figure 3) highlight the challenges of working with digital data streams. While prior research suggests that patients may overreport mental health symptoms to an app compared to in‐person visits due to anonymity, our results of the opposite suggest several possibilities. The fact that SZ scores were closer on the app compared to HC score suggests that patients may have found the app easier to use, that survey items were more relevant to their condition, or had more confidence in sharing information with the app. Although our study was not designed to assess why the control group reported symptoms with lower severity to the app, potential reasons include less trust in sharing information with apps which we have noted in prior research (Torous, Wisniewski, Liu, & Keshavan, 2018). This differential reporting of symptoms to technology based on illness, technology literacy, and other cultural factors remains a largely unexplored area around mobile mental health and warrants caution considering clinical use cases.

While our results utilized a sample with schizophrenia, given the higher prevalence of suicidal ideation that enabled us to explore correlations in latency, they are likely not specific to schizophrenia. The correlations between depression/suicide scores and latency suggest that the signal from this type of meta‐data is likely small and will require further higher risk samples to better characterize it. For instance, the lack of a significant correlation between score and latency in HC may be due to low power, with few scores greater than zero. In using app‐based reporting in our digital clinic for patients with varied disorders, we have noticed that latencies for depression survey items appear to correlate with patient‐reported outcomes more so than other items, a finding seen in results shown in Table 2. While we expected more symptom surveys that just depression to correlate with latency across both the SZ and HC cohorts, these results do support neurocognitive theories of depression. Although we are unable to assess if these latencies are more related to deficits in working memory, processing speed, or decision‐making—the potential of identifying proxies related cognitive control in depression via smartphone use opens a new source of easily accessible data for the field. The fact that there was no correlation between classical cognitive deficits in schizophrenia and latencies related to depression/suicide suggests an avenue for future research in specifying the cognitive domains this meta‐data may reflect (Appendix A).

While the control group did report a lower symptom burden than patients with schizophrenia, the control group still did share symptoms of depression not identifiable from the in‐person assessment alone. The PHQ‐9 as a diagnostic assessment for depression also has the ability to longitudinally assess individual symptoms of depression, which offers potential for personalized screening and risk assessment at a population level. While control data appears approximately zero for depression symptoms in the top panel of Figure 4, examining the latency data in the bottom panel of Figure 4 for controls presents new information that could potentially relate to latent symptoms or emerging risk. The fact that these latencies in healthy controls correlated with scores on all items of the PHQ‐9 except item 9 suggests value in further exploring this relationship and its potential to explore latent depression in otherwise healthy controls. One potential reason there was no correlation related to item 9 in HC is that the control population reported only three instances of elevated thoughts where item 9 was greater than zero across the entire study duration. In addition, latencies for item 9 were lower than latencies for items 1–8 in both SZ and HC, potentially suggesting separate analysis of this item from the rest of the PHQ‐9 items.

Like all studies, our results must be interpreted in light of several considerations. While we were able to control for age between the groups, we did not for education or race. Still, smartphone use is increasingly common among all people across the world regardless of education or race. While all study participants utilized their own smartphones, we also recognize that different smartphone models will have different latencies and that our results many potentially vary between devices. In this study, we aggregated individual data and examined group effects to minimize this bias which is inherent to any smartphone study where devices are not given to participants. Finally, we do not imply causality in our results but rather suggest latencies are a useful proxy that should be explored further.

## 4 CONCLUSION

As technologies like smartphones become tools to help assess suicide risk, using meta‐data related to response latencies offers a new source of information that possesses clinical correlations. Future research exploring the predictive validity of this meta‐data to identify latent depression, assess neurocognitive models of depression, predict risk based on longitudinal changes in latencies, and validate the correlations present here are each fruitful next steps for this work. To facilitate such work, we offer the smartphone app used in this study and resources to customize it for future work at digitalpsych.org/lamp/about.

## Supporting information


**Appendix** **A: PHQ‐9 item response value and latency after separating for cognition.**
SZ were separated into high and low cognition groups based on the median score for the in‐person BACS. Participants with cognitive scores in the lower 50% of the study sample took significantly (*p* < .05) more time on eight out of the nine items on the PHQ‐9 and responded with significantly different values on four out of the nine items as shown below.

## References

[mpr1825-bib-0001] Atkins, A. S. , Tseng, T. , Vaughan, A. , Twamley, E. W. , Harvey, P. , Patterson, T. , … Keefe, R. S. E. (2017). Validation of the tablet‐administered brief assessment of cognition (BAC app). Schizophrenia Research, 181, 100–106. 27771201 10.1016/j.schres.2016.10.010

[mpr1825-bib-0002] Barnett, I. , Torous, J. , Staples, P. , Sandoval, L. , Keshavan, M. , & Onnela, J.‐P. (2018). Relapse prediction in schizophrenia through digital phenotyping: A pilot study. Neuropsychopharmacology, 43(8):1660–1666. 10.1038/s41386-018-0030-z 29511333 PMC6006347

[mpr1825-bib-0003] Breier, A. , Schreiber, J. L. , Dyer, J. , & Pickar, D. (1991). National Institute of Mental Health longitudinal study of chronic schizophrenia. Prognosis and predictors of outcome. Archives of General Psychiatry, 48(3), 239–246. 10.1001/archpsyc.1991.01810270051007 1671741

[mpr1825-bib-0004] Deisenhammer, E. A. , Ing, C.‐M. , Strauss, R. , Kemmler, G. , Hinterhuber, H. , & Weiss, E. M. (2009). The duration of the suicidal process: How much time is left for intervention between consideration and accomplishment of a suicide attempt? The Journal of Clinical Psychiatry, 70(1), 19–24. 19026258

[mpr1825-bib-0005] Fann, J. R. , Berry, D. L. , Wolpin, S. , Austin‐Seymour, M. , Bush, N. , Halpenny, B. , … McCorkle, R. (2009). Depression screening using the patient health Questionnaire‐9 administered on a touch screen computer. Psycho‐Oncology, 18(1), 14–22. 10.1002/pon.1368 18457335 PMC2610244

[mpr1825-bib-0006] Hor, K. , & Taylor, M. (2010). Suicide and schizophrenia: A systematic review of rates and risk factors. Journal of Psychopharmacology (Oxford, England), 24(4 Suppl), 81–90. 10.1177/1359786810385490 PMC2951591 20923923

[mpr1825-bib-0007] Jo, H. , Na, E. , & Kim, D.‐J. (2018). The relationship between smartphone addiction predisposition and impulsivity among Korean smartphone users. Addiction Research and Theory, 26(1), 77–84.

[mpr1825-bib-0008] Kleiman, E. M. , Turner, B. J. , Fedor, S. , Beale, E. E. , Picard, R. W. , Huffman, J. C. , & Nock, M. K. (2018). Digital phenotyping of suicidal thoughts. Depression and Anxiety, 35(7), 601–608. 10.1002/da.22730 29637663

[mpr1825-bib-0009] Kroenke, K. , Spitzer, R. L. , & Williams, J. B. (2001). The PHQ‐9: Validity of a brief depression severity measure. Journal of General Internal Medicine, 16(9), 606–613. 10.1046/j.1525-1497.2001.016009606.x 11556941 PMC1495268

[mpr1825-bib-0010] Liu, G. , Henson, P. , Keshavan, M. , Pekka‐Onnela, J. , & Torous, J. (2019). Assessing the potential of longitudinal smartphone based cognitive assessment in schizophrenia: A naturalistic pilot study. Schizophrenia Research: Cognition, 17, 100144 10.1016/j.scog.2019.100144 31024801 PMC6476810

[mpr1825-bib-0012] Nock, M. K. , Borges, G. , Bromet, E. J. , Alonso, J. , Angermeyer, M. , Beautrais, A. , … Williams, D. (2008). Cross‐national prevalence and risk factors for suicidal ideation, plans and attempts. The British Journal of Psychiatry: The Journal of Mental Science, 192(2), 98–105. 10.1192/bjp.bp.107.040113 18245022 PMC2259024

[mpr1825-bib-0013] Palmer, B. A. , Pankratz, V. S. , & Bostwick, J. M. (2005). The lifetime risk of suicide in schizophrenia: A reexamination. Archives of General Psychiatry, 62(3), 247–253. 10.1001/archpsyc.62.3.247 15753237

[mpr1825-bib-0014] Pu, S. , Setoyama, S. , & Noda, T. (2017). Association between cognitive deficits and suicidal ideation in patients with major depressive disorder. Scientific Reports, 7(1), 11637 10.1038/s41598-017-12142-8 28912439 PMC5599636

[mpr1825-bib-0015] Rooksby, J. , Morrison, A. , & Murray‐Rust, D. (2019). *Student perspectives on digital phenotyping: The acceptability of using smartphone data to assess mental health* Proceedings of the 2019 CHI Conference on Human Factors in Computing Systems (pp. 425:1‐425:14). 10.1145/3290605.3300655

[mpr1825-bib-0016] Torous, J. , Larsen, M. E. , Depp, C. , Cosco, T. D. , Barnett, I. , Nock, M. K. , & Firth, J. (2018). Smartphones, sensors, and machine learning to advance real‐time prediction and interventions for suicide prevention: A review of current progress and next steps. Current Psychiatry Reports, 20(7), 51 10.1007/s11920-018-0914-y 29956120

[mpr1825-bib-0017] Torous, J. , Wisniewski, H. , Bird, B. , Carpenter, E. , David, G. , Elejalde, E. , … Keshavan, M. (2019). Creating a digital health smartphone app and digital phenotyping platform for mental health and diverse healthcare needs: An interdisciplinary and collaborative approach. Journal of Technology in Behavioral Science., 4, 73–85. 10.1007/s41347-019-00095-w

[mpr1825-bib-0018] Torous, J. , Wisniewski, H. , Liu, G. , & Keshavan, M. (2018). Mental health Mobile phone app usage, concerns, and benefits among psychiatric outpatients: Comparative survey study. JMIR Mental Health, 5(4), e11715 10.2196/11715 30446484 PMC6269625

[mpr1825-bib-0019] Westheide, J. , Quednow, B. B. , Kuhn, K.‐U. , Hoppe, C. , Cooper‐Mahkorn, D. , Hawellek, B. , … Wagner, M. (2008). Executive performance of depressed suicide attempters: The role of suicidal ideation. European Archives of Psychiatry and Clinical Neuroscience, 258(7), 414–421. 10.1007/s00406-008-0811-1 18330667

[mpr1825-bib-0020] Yan, T. , Ryan, L. , Becker, S. E. , & Smith, J. (2015). Assessing quality of answers to a global subjective well‐being question through response times. Survey Research Methods, 9(2), 101–109. 27398099 PMC4936784

